# Exploratory Analysis of Single-Gene Predictive Biomarkers in HERA DASL Cohort Reveals That C8A mRNA Expression Is Prognostic of Outcome and Predictive of Benefit of Trastuzumab

**DOI:** 10.1200/PO.18.00016

**Published:** 2018-11-01

**Authors:** Scooter Willis, Varvara Polydoropoulou, Yuliang Sun, Brandon Young, Zoi Tsourti, Dimitris Karlis, Bradley Long, Xiaoqian Lin, Stephanie Theel, Jennifer Carlson, Balazs Győrffy, Casey Williams, Mark Abramovitz, Urania Dafni, Mitch Dowsett, Brian Leyland-Jones

**Affiliations:** **Scooter Willis**, **Yuliang Sun**, **Brandon Young**, **Xiaoqian Lin**, **Stephanie Theel**, **Jennifer Carlson**, **Casey Williams**, **Mark Abramovitz**, and **Brian Leyland-Jones**, Avera Cancer Institute, Sioux Falls, SD; **Varvara Polydoropoulou**, **Zoi Tsourti**, **Dimitris Karlis**, and **Urania Dafni**, Frontier Science Foundation-Hellas; **Urania Dafni**, University of Athens, Athens, Greece; **Bradley Long**, Scripps Florida, Jupiter, FL; **Balazs Győrffy**, MTA-TTK Lendület Cancer Biomarker Research Group, Budapest, Hungary; and **Mitch Dowsett**, Royal Marsden Hospital, London, United Kingdom.

## Abstract

**Purpose:**

The Herceptin Adjuvant study is an international multicenter randomized trial that compared 1 or 2 years of trastuzumab given every 3 weeks with observation in women with human epidermal growth factor 2–positive (HER2+) breast cancer after chemotherapy. Identification of biomarkers predictive of a benefit from trastuzumab will minimize overtreatment and lower health care costs.

**Methods:**

To identify possible single-gene biomarkers, an exploratory analysis of 3,669 gene probes not expected to be expressed in normal breast tissue was conducted. Disease-free survival (DFS) was used as the end point in a Cox regression model, with the interaction term between C8A mRNA and treatment as a categorical variable split on the cohort mean.

**Results:**

A significant interaction between C8A mRNA and treatment was detected (*P* < .001), indicating a predictive response to trastuzumab treatment. For the C8A-low subgroup (mRNA expression lower than the cohort mean), no significant treatment benefit was observed (*P* = .73). In the C8A-high subgroup, patients receiving trastuzumab experienced a lower hazard of a DFS event by approximately 75% compared with those in the observation arm (hazard ratio [HR], 0.25; *P* < .001). A significant prognostic effect of C8A mRNA also was seen (*P* < .001) in the observation arm, where the C8A-high group hazard of a DFS event was three times the respective hazard of the C8A-low group (HR, 3.27; *P* < .001). C8A mRNA is highly prognostic in the Hungarian Academy of Science HER2+ gastric cancer cohort (HR, 1.72; *P* < .001).

**Conclusion:**

C8A as a single-gene biomarker prognostic of DFS and predictive of a benefit from trastuzumab has the potential to improve the standard of care in HER2+ breast cancer if validated by additional studies. Understanding the advantage of overexpression of C8A related to the innate immune response can give insight into the mechanisms that drive cancer.

## INTRODUCTION

Breast cancer is the leading cause of cancer death among women worldwide.^1^ It is a heterogeneous disease, so to maximize benefit to the patient by individualizing treatment regimens, clinicians need a sound classification system. Clinicopathologic variables, including the estrogen receptor 1, progesterone receptor, and human epidermal growth factor receptor 2 (HER2), are used to classify tumors as hormone positive, HER2 positive (HER2+), or triple negative and to guide treatment decisions. Breast cancer can be further classified through gene expression profiling into PAM50 (Prosigna, distinct subtypes (luminal A, luminal B, HER2-enriched, basal-like, and normal-like^2^), with significant differences in prognosis and response to treatment.^3^ Oncotype DX^4^ (Genomic Health, Redwood City, CA) and MammaPrint^5^ (Agendia, Irving, CA) are commercially available gene expression assays that determine a risk score that can be used to tailor treatment. PAM50, Oncotype DX, and MammaPrint were developed using retrospective analysis of mRNA gene expression data from breast cancer cohorts.

Identification of single genes or gene signatures that can be used to classify a breast cancer tumor as a subtype with known treatment strategies can improve outcomes and minimize unnecessary treatment. Ultimately, biomarkers predictive of response to a specific treatment are needed to improve the standard of care. The identification that 15% to 25% of breast cancer tumors overexpress the HER2 protein, a transmembrane tyrosine kinase that regulates growth and cell survival, resulted in the development of monoclonal antibody therapy that targets HER2. The Herceptin Adjuvant (HERA; Breast International Group [BIG] 01-01) clinical trial showed definitively that trastuzumab, a monoclonal HER2 antibody, is an effective treatment strategy for HER2+ breast cancer and has changed the standard of care.^6^ The HERA trial compared 1 year of trastuzumab treatment versus observation and found a hazard ratio (HR) of 0.76 (95% CI, 0.67 to 0.86; *P* < .001) for disease-free survival (DFS) and 0.76 (95% CI, 0.65 to 0.88; *P* < .001) for overall survival (OS), with an 8-year median follow-up and 52% of patients from the observation group crossing over to trastuzumab therapy.^7^ The determination of predictive biomarkers that identify an HER2+ subtype that will benefit from the addition of trastuzumab despite the high survival percentage treated with chemotherapy for early HER2+ breast cancer^8^ will reduce overtreatment and become a more cost-effective treatment strategy.^9^

The HERA cDNA-mediated annealing, selection, extension, and ligation (DASL) cohort, which consisted of 610 HERA formalin-fixed, paraffin-embedded (FFPE) samples from the TransHERA cohort profiled on the Illumina DASL platform (San Diego, CA), was used to conduct an exploratory analysis of 3,669 mRNA gene probes not expected to be expressed in normal breast tissue^10^ to identify genes that are possibly predictive of benefit from trastuzumab. The exploratory analysis identified C8A, a member of the membrane attack complex and part of the innate immune system, which was prognostic of outcome in the observation arm and predictive of benefit from trastuzumab. C8A inserts into the membrane of the target cell and binds with multiple copies of the pore-forming C9, leading to cell lysis. From the GeneAtlas, *C8A* mRNA is highly expressed only in liver tissue, and the Cancer Cell Line Encyclopedia indicates a wide range of *C8A* mRNA expression in cancer cell lines.

In this study, we explored C8A mRNA as a predictive biomarker in the HERA DASL cohort, as a prognostic marker of outcome in gastric cancer, and as a possible indicator of a specific HER2 subtype. We also characterized C8A protein expression in stable cancer cell lines.

## METHODS

### Study Population

The HERA trial was an international, intergroup, open-label, phase III randomized study.^5^ A total of 5,102 women with HER2+ primary breast cancer (after a minimum of four courses of standard chemotherapy) were enrolled and assigned randomly to one of the following three treatment arms: observation (no trastuzumab) and 1 or 2 years of adjuvant trastuzumab administered intravenously every 3 weeks. Before enrollment, patients gave written informed consent to participate in the study. In addition, they had the option to donate their breast tumor and serum for future research purposes. An interim analysis^5^ showed that patients assigned to the 1-year trastuzumab arm experienced a significantly lower hazard of a DFS event than those in the observation arm. This finding resulted in a protocol amendment that allowed for observation patients to selectively crossover to trastuzumab treatment (1 or 2 years), with the restriction of being alive and disease-free as of May 16, 2005. The intention-to-treat population comprised 5,099 women (three patients were excluded because of missing informed consent forms). HERA patients gave consent to donate breast tissue for additional research purposes (TransHERA studies).

### HERA DASL Cohort

TransHERA was set up as the translational aspect of the HERA trial, although it was not initially integrated into the main study. The TransHERA tissue resource consisted of 1,203 blocks that originated from 15 countries and were processed at Royal Marsden Hospital in the United Kingdom. In addition, an extra 600-μm core was taken from each block. Sample RNA was extracted using the ExpressArt FFPE RNAready isolation kit (AmpTec, Hamburg, Germany), which is optimized for isolation of total RNA specifically from FFPE tissue samples, and eluted into a volume of 20 μL. A single 600-μm core was used for extraction of RNA for gene expression analyses. The concentration of the RNA was determined using a spectrophotometer (NanoDrop ND-1000; Thermo Fisher Scientific, Waltham, MA). A total of 751 of the 828 available HERA FFPE 600-μm core samples had quality RNA available for the gene expression analyses.

For this study, molecular profiling was performed on 100 ng of RNA using an Illumina whole-genome DASL assay specifically designed to capture mRNA expression levels from archived FFPE tissue samples. The whole-genome DASL method uses biotinylated random nonamer and oligo (dT) primers to convert input RNA to cDNA. The biotinylated cDNA is then immobilized to a streptavidin-coated solid support and annealed to a DASL assay pool of gene-specific oligonucleotides for extension and ligation followed by polymerase chain reaction amplification with a biotinylated and a fluorophore-labeled universal primer. Finally, the single-stranded polymerase chain reaction products were eluted and hybridized to the Illumina HumanHT-12 v3 Expression BeadChip. Each oligonucleotide probe is represented, on average, by 30 beads per hybridized sample. Control (liver and brain) RNA samples were included for each processed batch of 48 samples to ensure that RNA processing was successful and for quality control and normalization of data between assay batches. Illumina probe-gene annotations were programmatically mapped to the current Human Genome Organisation gene annotation. From the 751 samples processed, 610 passed Illumina quality control metrics and were used to establish the HERA DASL cohort. Normalization of the gene expression data was performed by the cubic spline method. Cubic spline has been shown to combine the positive effects of quantile normalization and to avoid the drawbacks of discontinuous mapping of intensity values and no-rank preservation.^11^

### Analysis

Information from the HERA database, with a clinical cutoff date of April 12, 2012, and 8 years of median follow-up, was used in the current study.^7^ The primary end point was DFS, defined as the time from random assignment to first occurrence of any of the following DFS events: recurrence of breast cancer at any site; the development of ipsilateral or contralateral breast cancer, including ductal carcinoma in situ but not lobular carcinoma in situ; second nonbreast malignant disease other than basal-cell or squamous-cell carcinoma of the skin or carcinoma in situ of the cervix; or death as a result of any cause without documentation of a cancer-related event.

### Exploratory Analysis of Possible Predictive Biomarkers

**Fig 1.**
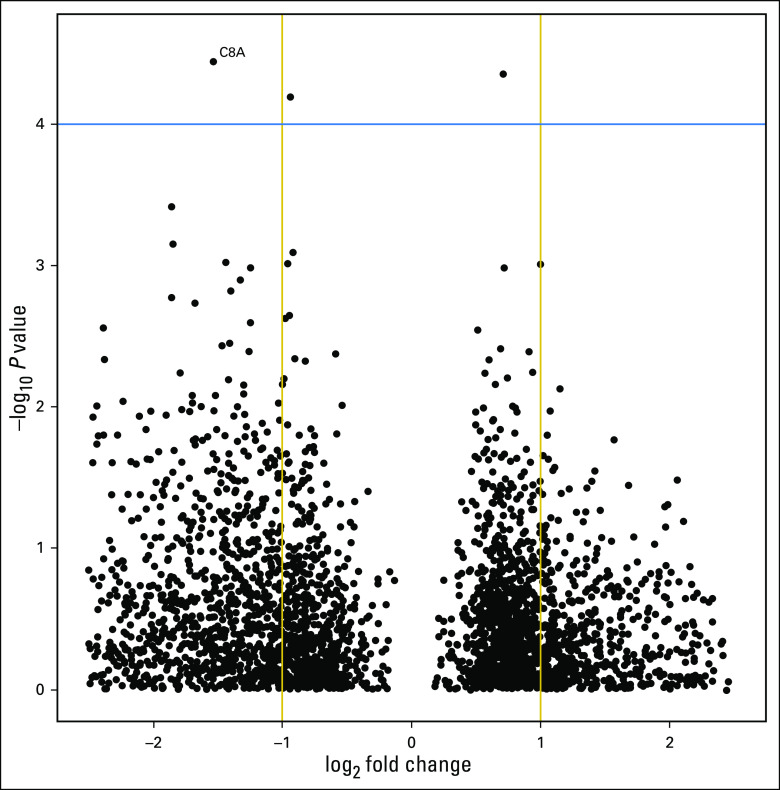
Exploratory analysis of treatment interaction *P* value as a categorical variable split on probe mRNA expression of 3,669 gene probes in the Herceptin Adjuvant cDNA-mediated annealing, selection, extension, and ligation cohort that have zero expected expression in normal breast tissue. The *y*-axis shows the −log_10_ interaction *P* value where, in this example, C8A is 3.63 × 10^5^. The *x*-axis shows the log_2_ fold change where the *P* value represents the expression difference between the log-mRNA expression for the 25th and 75th percentiles. The negative fold change indicates a hazard ratio < 1.0 or that high expression predicts benefit from trastuzumab. For C8A mRNA expression, the difference between the 25th and 75th percentile was −1.54.

In this exploratory analysis, 3,669 gene mRNA probes on the Illumina HumanHT-12 v3 array, which are based on normal breast tissue mRNA expression profiles not expected to be expressed in breast tissue,^10^ were used to conduct an exploratory analysis of possible predictive biomarkers.^1^ Each probe was used in a Cox proportional hazards regression predictive model as a categorical variable bifurcated on the mean of gene mRNA expression. The mean of gene mRNA—not the median—was used to capture the inflection point of a high- versus low-expression profile. Use of the median would have compared half of the cohort at a cut point that is not relevant to the biomarker expression level. In this particular case, approximately 75% of the HERA DASL cohort had no expression of *C8A* mRNA, so the mean establishes an unbiased cut point at the transition from low- to high-expression of *C8A* mRNA. The list of probes ranked by interaction *P* value were then used to identify C8A as a gene of interest because it is not normally expressed in breast tissue and is a member of the membrane attack complex and part of the innate immune system. A volcano plot of the exploratory analysis is shown in Figure 1. The complete list of possible predictive genes in the HERA DASL cohort is not provided given the complexities of reviewing the biologic implications of each gene and of finding supporting evidence that the genes are informative of outcome in other established cohorts.

### Statistical Analysis of the C8A HERA DASL Cohort

The observation that the *C8A* biomarker was predictive of response to trastuzumab treatment in the exploratory analysis was primarily explored through an intention-to-treat analysis; that is, crossover was not taken into account, and patients were analyzed according to their initial treatment assignment (the 1-year and 2-year trastuzumab arms were combined into a single arm referred to as the combined trastuzumab arm). To circumvent the selective crossover effect of early events, censored and inverse probability weighted (IPW) analyses also were performed. In the early events analysis, only early disease-defining events were taken into account (ie, at 2.2 years of median follow-up time), when almost no patient had switched from observation to trastuzumab. In the censored analysis, all patients under observation who switched to trastuzumab were censored the day they received active treatment. Finally, in the IPW analysis, the real treatment effect was assessed by re-creating the population that would have been observed without crossover through statistical modeling and weight assignment.

The representativeness of the HERA DASL cohort with respect to the overall HERA population was explored by comparing basic patient and tumor baseline characteristics as well as outcome using Fisher’s exact and Mantel-Haenszel (log-rank) tests at the 5% level of significance. Balance of baseline characteristics between the two treatment groups was assessed the same way. The distribution of *C8A* is presented through descriptive statistics and histograms. A cutoff value of 600 (close to the cohort mean) was considered to categorize the *C8A* biomarker as low or high. Cox proportional hazards regression models were used to model DFS and to obtain HRs corresponding 95% CIs. The interaction term between treatment and *C8A* was of main interest. *C8A* entered the model as both a continuous and a categorical variable (low/high). Cox models were adjusted for several clinicopathologic factors: age, pathologic tumor size, progesterone receptor local, estrogen receptor local, tumor grade, menopausal status, nodal status, prior (neo)adjuvant chemotherapy, Eastern Cooperative Oncology Group performance status, race, and region. The final model was selected through the backward elimination procedure, with a removal criterion of 10%. Observed differences in hazard were depicted graphically through the Kaplan-Meier method. Median follow-up time was estimated using the reverse-censoring method for OS. The weights for the IPW analysis were estimated using a time-varying covariate and the following two baseline covariates: age at study entry and prior (neo)adjuvant chemotherapy. Follow-up time of each eligible patient was divided into 1-month intervals, and the time-varying covariate was estimated as the within-interval cumulative time of eligibility to cross over.

### Prognostic HER2+ Gastric Cancer Cohort

We used the publicly available data from the Hungarian Academy of Science (HAS) gastric cancer cohort to further explore *C8A* as a possible biomarker in HER2+ gastric cancer.^12^ The HAS gastric cancer cohort is a collection of publicly available cohorts with outcome data profiled on the Affymetrix platform (Santa Clara, CA). The original cell values from each cohort were combined and renormalized^13,14^ and have been made available for prognostic biomarker analysis through the Kaplan-Meier Plotter Web site (http://kmplot.com). The HAS gastric cancer cohort (N = 882) with OS as outcome consisting of several cohorts (GSE14210, GSE15459, GSE22377, GSE29272, GSE51105, and GSE62254). HER2 status was determined using the GeneChip probe set 216836_s_at as described previously,^15^ which resulted in an HER2+ gastric cancer cohort (n = 344).

### C8A Phenotype

To identify possible phenotypic differences in the HERA DASL cohort between HER2+ tumors with high *C8A* and low *C8A* expression, Gene Set Enrichment Analysis (GSEA)^16^ was performed. Enriched gene sets with false discovery rate (FDR)–adjusted *P* values < 5% were considered statistically significant. Molecular Signature Database (version 4.0) gene sets tested included immunologic signatures (n = 1,910), canonical pathways (n = 1,320), molecular gene ontology terms (n = 1,454), microRNA (n = 221), chromosome positions (n = 326), transcription factors (n = 615), chemical genetic perturbations (n = 3,402), and oncogenic signatures (n = 189).

### *C8A* mRNA Normal and Cancer Tissue Expression Profile

The GTEx Web portal^17^ was used to generate a figure for the C8A mRNA expression in normal tissues. Figures that illustrate *C8A* mRNA expression from cancer cell lines were generated using the Cancer Cell Line Portal.^18^ The Cancer Cell Line Encyclopedia was used to determine the *C8A* mRNA mean and standard deviation in 1,036 cancer cell lines. Cancer cell lines with *C8A* > 2 standard deviations from the mean were listed. Cancer cell lines with known high *C8A* mRNA expression and available in the laboratory were used as controls to validate C8A immunohistochemistry (IHC) staining. The C8A IHC work was used to establish that the *C8A* mRNA and resulting protein are attributes or features of the cancer cell line that possibly give that cancer cell line a fitness advantage as opposed to measuring an unknown source of *C8A* mRNA given tumor heterogeneity and a possible innate immune response.

### C8A IHC Staining

The Complement C8A/B/G mouse monoclonal antibody (Cat. No. NB100-64340; Novus Biologicals, Littleton, CO) was used to stain for C8A protein expression in normal liver and an ovarian cancer cell line (OV90) as a positive control. Tumor cell lines, hepatocellular carcinoma (TMA809), lung cancer (DMS454), triple-negative breast cancer (MDA-231), HER2 normal breast cancer (MDA-175), and HER2+ breast cancer (SK-BR-3) were used for C8A IHC staining. Analyses were carried out in SAS 9.3 statistical software (SAS Institute, Cary, NC), GSEA version 2.07^16^ (Broad Institute, Cambridge, MA), the GTEx Web portal,^17^ the Cancer Cell Line Portal,^18^ the Cancer Cell Line Encyclopedia,^19^ and the Kaplan-Meier Plotter Web interface.^13,14^

## RESULTS

### Study Population

The HERA DASL cohort consisted of 610 patients with complete information on the C8A biomarker as well as on outcome, with a median follow-up time of approximately 8 years (interquartile range, 7.1 to 8.5 years). With regard to baseline characteristics, the HERA DASL cohort is representative of the HERA population, with the exception of race and region (Data Supplement). With respect to outcome, the percentage of DFS events was similar for both cohorts (28.5% for the HERA DASL cohort and 29.8% for the remainder of HERA patients), and no significant difference was observed (HR_comb. trast._ *_v_*_obs._, 0.69 [95% CI, 0.51 to 0.94] for the HERA DASL cohort and 0.77 [95% CI, 0.69 to 0.86] for the remainder of HERA patients; *P* = 0.18; Data Supplement).

**Fig 2.**
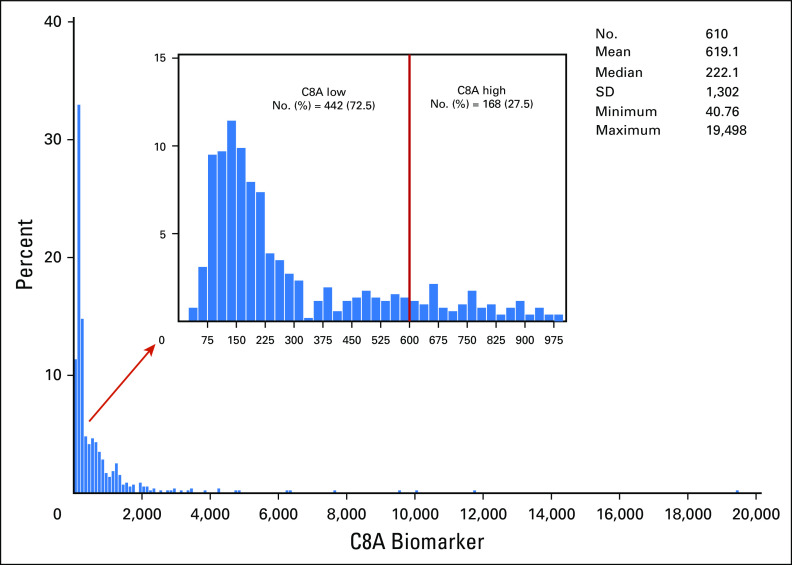
*C8A* mRNA expression in the Herceptin Adjuvant cDNA-mediated annealing, selection, extension, and ligation cohort split on the cohort mean. The mRNA expression profile suggests that for approximately 70% of the human epidermal growth factor 2–positive cohort, *C8A* mRNA was not expressed, and 30% had high expression of *C8A* mRNA. SD, standard deviation.

Of the 610 patients, 199 were randomly assigned to the observation arm and 411 to the combined trastuzumab arm. The corresponding observed DFS events were 66 and 108, respectively. Characteristics were well balanced between the two treatment groups (all *P* values > 5%; Data Supplement). *C8A* biomarker distribution is shown in Figure 2. *C8A* was overexpressed in 168 patients (27.5%), and no difference in its distribution was detected with respect to treatment arms (*P* = .21).

### C8A mRNA Prognostic and Predictive in HERA DASL Cohort

**Fig 3.**
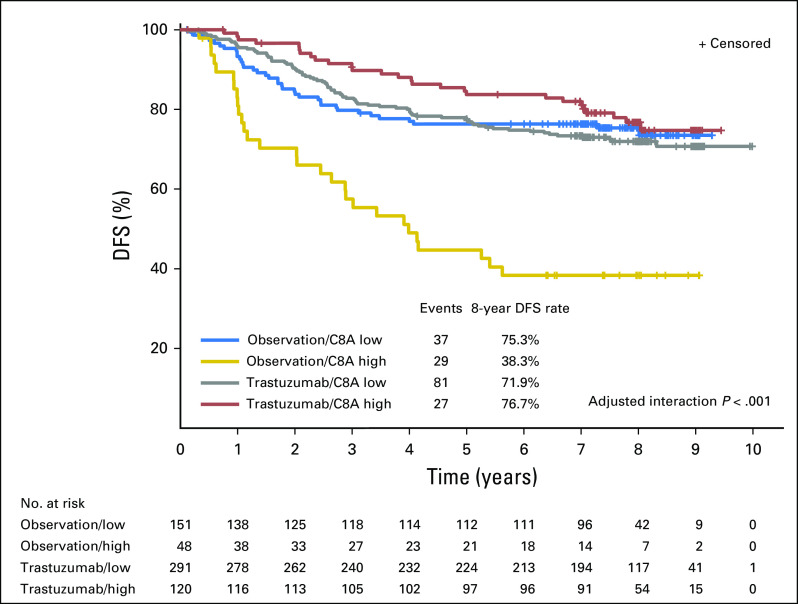
Kaplan-Meier curve for disease-free survival (DFS) in the Herceptin Adjuvant cDNA-mediated annealing, selection, extension, and ligation cohort, where the C8A mRNA split on the mean represents C8A-low and C8A-high categories. A statistically significant interaction between C8A mRNA and treatment was detected (*P* < .001), which indicates that C8A mRNA is predictive of response to trastuzumab treatment. For the C8A-low patient subgroup (mRNA expression lower than the cohort mean), no significant treatment benefit was observed (*P* = .73). On the other hand, for the C8A-high subgroup, patients in the trastuzumab arm experience a lower hazard of a DFS event by approximately 75% compared with patients in the observation arm (hazard ratio, 0.25; 95% CI, 0.15 to 0.43; *P* < .001). A significant prognostic effect of C8A mRNA also was observed (*P* < .001) in the observation arm, where in the C8A-high subgroup, the hazard of a DFS event was three times that in the C8A-low subgroup (hazard ratio, 3.27; 95% CI, 2.01 to 5.32, *P* < .001).

A significant interaction between categorical *C8A* and treatment was observed (*P* < .001; Table 1). Patients classified as *C8A* high derived a statistically significant treatment benefit, with the hazard of a DFS event being lower by 75% for patients treated with trastuzumab than in those in the observation arm (HR_comb. trast._ *_v_*_obs._, 0.25; 95% CI, 0.15 to 0.43; *P* < .001). On the other hand, no significant treatment benefit was detected in the *C8A*-low patient subgroup (*P* = .73). In addition, a significant prognostic *C8A* effect was observed (*P* < .001). More specifically, patients with *C8A* overexpression experienced three times the respective hazard of a DFS event compared with those without (HR_C8A high_ *_v_*_low_, 3.27; 95% CI, 2.01 to 5.32; *P* < .001). Both the predictive and the prognostic effects of *C8A* remained significant when adjusted for nodal status and region (C8A-high group: HR_comb. trast._ *_v_*_obs._, 0.23 [95% CI, 0.13 to 0.39; *P* < .001]; C8A-low group: *P* = .97; observation arm: HR_C8A high_ *_v_*_low_, 3.06 [95% CI, 1.86 to 5.03]; Table 2; Fig 3). When the *C8A* biomarker entered the model as a continuous variable, a statistically significant treatment-by-biomarker interaction was detected only in the multivariable model (*P* = .0062 adjusted for estrogen receptor local, tumor grade, nodal status, and region; Data Supplement), whereas no significant predictive effect was observed in the simple model (*P* = .07). These findings were verified by the early event, censored, and IPW analyses.

**Table 1.**
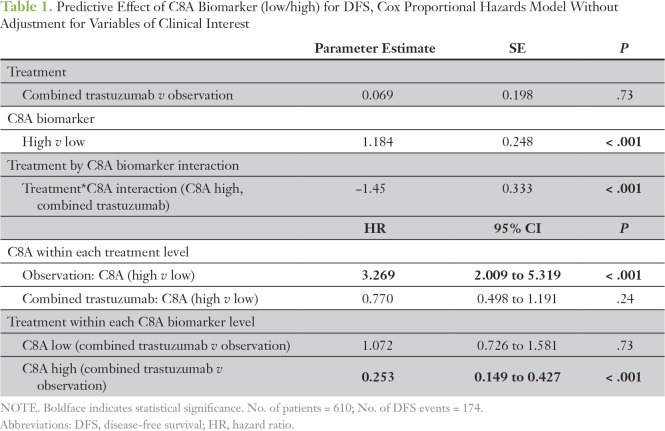
Predictive Effect of C8A Biomarker (low/high) for DFS, Cox Proportional Hazards Model Without Adjustment for Variables of Clinical Interest

**Table 2.**
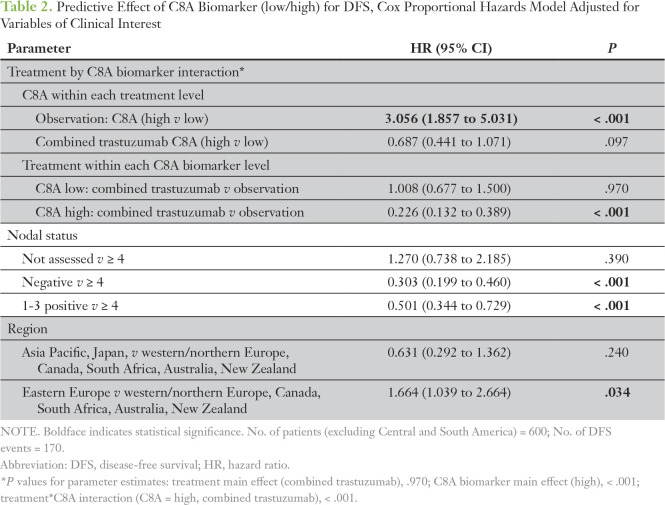
Predictive Effect of C8A Biomarker (low/high) for DFS, Cox Proportional Hazards Model Adjusted for Variables of Clinical Interest

### *C8A* mRNA Prognostic HAS HER2+ Gastric Cancer Cohort

On the basis of the publicly available data from the HAS gastric cancer cohort, a significant prognostic effect of *C8A* mRNA on OS was observed (HR, 1.72; 95% CI, 1.32 to 2.23; *P* < .001; Data Supplement). The corresponding *C8A* mRNA expression profile in the HAS HER2+ gastric cancer cohort is presented in the Data Supplement. For the *C8A* low-expression group, *C8A* mRNA was minimal or had no expression similar to the expression profile of *C8A* in the *C8A* HERA DASL cohort.

### *C8A* Phenotype

**Fig 4.**
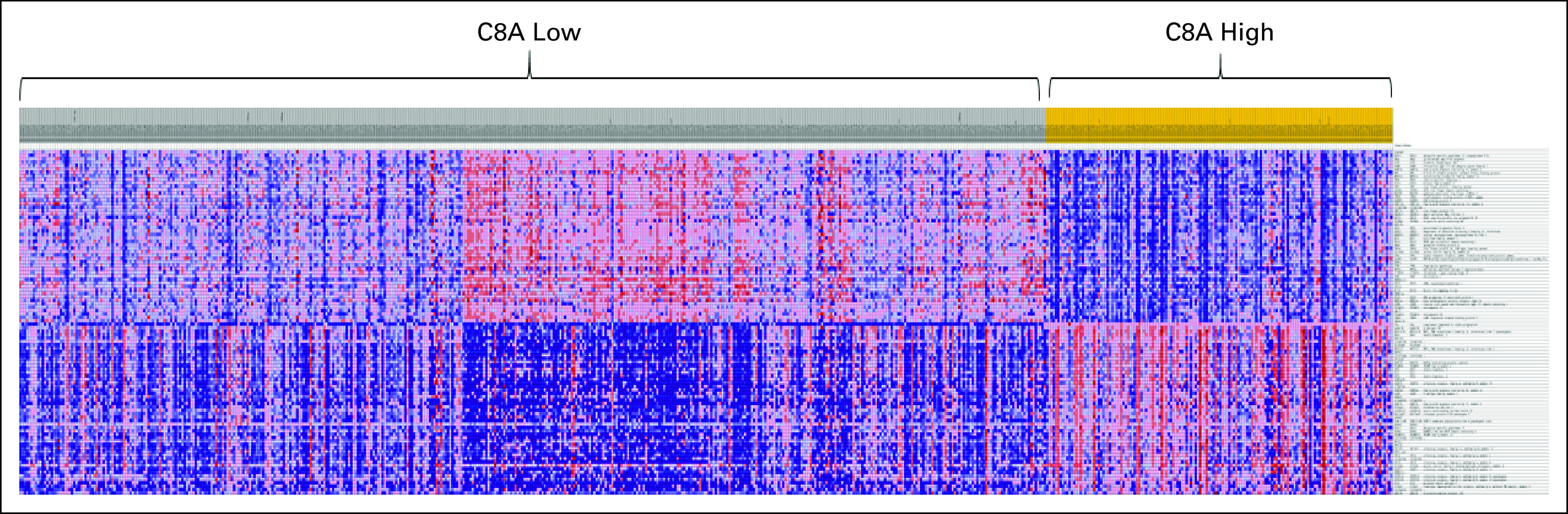
GSEA histogram comparing Herceptin Adjuvant cDNA-mediated annealing, selection, extension, and ligation cohort samples by *C8A* mRNA low- versus high-expression status. The immune gene list from Molecular Signature Database (version 4.0) on the right represents the top 50 enriched immune genes in *C8A*-low versus *C8A*-high samples and the top 50 enriched immune genes in *C8A*-high versus *C8A*-low samples. A detailed list of enriched genes is provided in the Data Supplement.

In the *C8A* low-expression group, 741 of the 1,910 gene sets (FDR *P* < .05) had higher expression of mRNA for genes associated with immunologic response. The *C8A* high-expression group had no statistically significant (*P* < .01) upregulated immunologic gene sets. No other gene sets with FDR *P* < .05 were found in the collection of Molecular Signature Database gene sets tested. The *C8A* phenotype is illustrated in the GSEA histogram shown in Figure 4, with the top 50 genes enriched in the *C8A*-low group versus the top 50 genes enriched in the *C8A*-high group. C8A is part of the innate immunity and membrane attack complex and clearly differentiated the enrichment of gene sets associated with immunologic response.

### *C8A* mRNA Expression

The *C8A* mRNA on/off expression profile in our analysis cohort suggests that C8A may be expressed in other cancer cell lines. On the basis of the results presented in the Data Supplement, we see that *C8A* is highly expressed in distinct ovarian, liver, stomach, pancreatic, lung, and hematopoietic/lymphoid cancer types. The expression of *C8A* in cancer cell lines compared with copy number suggests that *C8A* expression is not the result of chromosomal aberrations (Data Supplement). To verify that *C8A* mRNA results in C8A protein expression, C8A antibody was used to stain available cancer cell lines, and the results are the Data Supplement.

## DISCUSSION

Identification of single genes or gene signatures that can be used to classify a breast cancer tumor as a subtype with known treatment strategies can improve outcomes and minimize unnecessary treatments. Ultimately, biomarkers that are predictive of response to a specific treatment are needed to improve the standard of care.

Preliminary results of an exploratory analysis of possible predictive biomarkers that are not expected to be expressed in normal breast tissue suggest that *C8A* is prognostic of DFS and predictive of trastuzumab benefit in the HERA DASL cohort but requires additional validation. The highly prognostic effect of *C8A* mRNA in the HAS gastric cancer cohort suggests that C8A gives the tumor a survival advantage. In addition, low expression of *C8A* indicates an enrichment of immunologic genes, which suggests that high *C8A* mRNA is a possible negative indicator of immune response. The Cancer Cell Line Encyclopedia data show that DMS454_LUNG, OV90_OVARY, and SNU719_STOMACH are examples that have high expression of C8A and that C8A is a feature of those immortal cancer cell lines.

C8A protein is a critical component of the membrane attack complex that inserts itself into the outer membrane of target cells and anchors the recruitment of C9 proteins to form a pore that leads to cell lysis and death.^20,21^ The membrane attack complex is a component in the complement system, which is part of the innate immune system found in plant and animals and evolved before adaptive immunity.^22^ The innate immune system can work independently of or be triggered by the adaptive immune system and plays a role in the monitoring of host cells that are damaged or have died and should be cleared.^23^ The complement system proteins are synthesized by the liver and normally circulate in the blood until stimulated by complement activation. No other members of the membrane attack complex have been shown to be prognostic or predictive of outcome. The finding that *C8A* mRNA predicts response to treatment with an HER2-targeting antibody that triggers an adaptive immune response and is highly prognostic strongly suggests that the C8A protein may provide a tumor survival advantage that, like *PD-L1*, suppresses the immune response.^24^ This also is supported by the GSEA analysis, which showed that a large number of immune-related gene sets are enriched in the *C8A*-positive tumors compared with the *C8A*-negative tumors in the HERA DASL cohort.

Preliminary results suggest that *C8A* is expressed in a range of immortal cancer cell lines and offers a survival advantage in HER2+ breast cancer and HER2+ gastric cancer. Molecular characterization of *C8A* high HER2+ tumors may indicate that it is a new cancer phenotype that can escape the immune response and become an important mechanism in cancer that affects survival. This possibility merits assessment of its validity in other cohorts of trastuzumab-treated malignancies.
